# Accelerometer-based navigation *vs*. conventional techniques for total knee arthroplasty (TKA): a systematic review and meta-analysis of randomized controlled trials

**DOI:** 10.1186/s42836-022-00135-6

**Published:** 2022-09-02

**Authors:** Juntan Li, Yuqi Zhang, Xiang Gao, Tianxu Dou, Xu Li

**Affiliations:** 1 Department of Orthopedics, The First Hospital of China Medical University, No.155 Nanjing North Street, Shenyang, Liaoning Province 110001 People’s Republic of China; 2 School of Public, Health China Medical University, No.77 Puhe Road, Shenyang, Liaoning Province 110122 People’s Republic of China; 3 Department of Orthopedics, The Fourth Hospital of China Medical University, Chongshan East Road, Shenyang, Liaoning Province 110000 People’s Republic of China

**Keywords:** Total knee arthroplasty, Accelerometer-based navigation, Systematic review, Meta-analysis

## Abstract

**Background:**

The aim of the study was to determine whether accelerometer-based navigation (ABN) can improve radiological and functional outcomes during total knee arthroplasty (TKA) compared with conventional techniques (CONV).

**Method:**

We comprehensively searched the PubMed, Embase, Web of Science, Cochrane Library, and Clinical Trials databases. Only randomized controlled trials were selected for meta-analysis and, ultimately, 10 studies were included.

**Results:**

The 10 studies involved 1,125 knees, of which 573 were in the ABN group and 552 in the CONV group. The results demonstrated that ABN significantly reduced the number of outliers for mechanical alignment (MA) (RR: 0.38, 95% CI: 0.27 to 0.54, *P* < 0.00001, I^2^ = 45%), achieving more accurate MA (RR: –0.78, 95% CI: –0.93 to –0.62, *P* < 0.00001, I^2^ = 76%). The results revealed that there was no significant difference in duration of surgery between the ABN and CONV groups (MD: –0.2, 95% CI: –1.45 to 1.05, *P* = 0.75, I^2^ = 48%). There was less blood loss through the use of ABN (SMD: –0.49, 95% CI: –0.93 to –0.06, *P* = 0.03, I^2^ = 75%). However, ABN group didn’t show better knee function (SMD: 0.13, 95% CI: –0.07 to 0.33, *P* = 0.20, I^2^ = 0%), though the incidence of overall complications was significantly lower (RR: 0.69, 95% CI: 0.50 to 0.95, *P* = 0.02, I^2^ = 0%).

**Conclusions:**

The present meta-analysis demonstrated that ABN was superior to CONV in restoring MA of the lower limb. In addition, ABN reduced the loss of blood and the duration of surgery was not prolonged. However, patient-reported outcome measurements (PROMs) were not improved.

## Introduction

Total knee arthroplasty (TKA) is the treatment of choice for end-stage knee diseases. It is projected that the annual number of primary TKA procedures in the US in 2030 and 2040 will be 182% and 401% greater than levels reported in the national inpatient sample (NIS) data [1]. However, approximately 20% of patients do not have satisfactory knee function following TKA [2]. A number of studies have reported that mechanical alignment (MA) of the lower limb significantly influences the outcome of TKA [3–5], and, therefore, accurate restoration of MA is important in TKA. Coronal MA of greater than 3 degrees has been shown to result in a higher rate of failure [6, 7], and so postoperative MA that was varus or valgus by more than 3 degrees was defined as an ‘outlier’ following TKA. Liu *et al.* reported that the postoperative failure rate in knees with varus alignment was significantly higher than those with neutral alignment, resulting in shorter survival after TKA [8]. Koen *et al.* demonstrated that out-of-range MA, especially varus, led to higher tibial component migration in a 5-year follow-up examination [9]. Various new methods and instruments have been developed to reduce MA outliers during TKA.

Conventional TKA techniques (CONV) are generally based on an intramedullary guide for femoral bone cutting with extramedullary tibial bone cutting. Computer-assisted navigation (CAN) depends on optical navigation for accurate restoration of MA, but the prolonged duration of surgery, the cost of hardware and software, additional pin sites, and the steep learning curve have limited its acceptance in surgical practice [10, 11]. More recently, robotic assistance has been at the forefront of surgical innovation in TKA, such as MAKO and ROBODOC [12], but these robots are expensive [13]. Accelerometer-based navigation (ABN), introduced in the 2010s, provides levels of accuracy similar to that of CAS and robots in TKA, but at a lower cost and with high portability [14, 15]. Typical devices include KneeAlin (OrthAlign, Aliso Viejo, CA), iASSIST (Zimmer, Warsaw, IN, USA), and i-JOIN (i-JOIN, Shanghai, China). However, the results of previously published studies were highly variable, so this meta-analysis was performed to update the evaluation of the clinical benefits of ABN.

Comparisons of ABN and CONV in TKA have already been the subject of review and meta-analysis, but with studies included being of heterogenous quality [16]. In the present review and meta-analysis, only randomized controlled trials (RCTs) were included so that the comparison of ABN and CONV in TKA would be more convincing [17–26]. The aim of the review was to determine whether ABN is able to reduce the proportion of outliers, as radiologically assessed, whether ABN is able to achieve superior surgery-related outcomes, and whether patient-reported outcome measurements (PROMs) are greater with the application of ABN.

## Materials and methods

### Search strategy and study selection

The review and meta-analysis were conducted in accordance with the Preferred Reporting Items for Systematic Reviews and Meta-Analyses (PRISMA) checklist and guidelines [27]. The protocol for this review was posted online at the PROSPERO International Prospective Register of Systematic Reviews (https://www.crd.york.ac.uk/PROSPERO/) with registration number CRD42021278442. A comprehensive search was separately conducted by two reviewers (Li and Zhang) to ensure accuracy. The following terms were used when searching the PubMed, Embase, Web of Science, Cochrane Library, and Clinical Trials databases: “arthroplasty, replacement, Knee” and “accelerometer based navigation” or “portable navigation”, for articles published between January 2000 and September 2021, with no restriction to publication language. Relevant studies were identified after reading the titles and abstracts of each article, after which the full text was assessed to confirm whether the article should be included. All disagreements were resolved through discussion between reviewers.

### Eligibility criteria

Inclusion and exclusion criteria established for the review were based on a PICOS design (patient, intervention, control, outcomes, and study). Only randomized controlled trials (RCTs) that directly compared ABN with CONV in TKA and reported at least one of the following outcomes were included: outliers for mechanical alignment (MA), coronal femoral angle (CFA) of the prosthesis, coronal tibial angle (CTA) of the prosthesis, tibial slope (TS), duration of surgery, blood loss, and patient-reported outcome measurements (PROMs). Non-randomized controlled trials and reviews were excluded, as were unicompartmental knee arthroplasty-related studies.

### Quality assessment

Each article was evaluated by two authors (Li and Zhang). If a discrepancy between the reviewers arose, a discussion was held among all authors to reach a consensus. Risk of bias in the included studies was assessed using the Cochrane risk of bias tool to determine whether the results were affected, regarding the following parameters: randomization procedure, allocation concealment, blinding of patients and surgeons, and blinding of outcome assessors, selective outcome reporting, incomplete outcome data and other biases. Two authors independently judged whether each constituted a high, low, or unclear risk of bias [28].

### Data extraction and analysis

The primary outcome of the meta-analysis was the determination of several radiographic parameters: MA and outliers of the MA, outliers of CFA and CTA and outliers of TS. Secondary outcomes included blood loss and duration of surgery, used to evaluate any harm caused by the surgery and the time required for performing ABN. Other outcomes included knee function, which was evaluated using PROMs and surgical complications, such as deep vein thrombosis (DVT), fracture during surgery, infection, or death. Two authors independently extracted data from each study included in the review, after which the data were recorded in Microsoft Excel. Study information, such as author, year of publication, country, and journal, was extracted initially, after which the characteristics of the participants, such as sex, age, body mass index (BMI), and type of ABN were extracted.

### Statistical analysis

For dichotomous outcomes, such as the number of outliers and adverse events, the risk ratio (RR) and the associated 95% confidence interval (CI) were calculated to obtain the differences between the techniques for each study. Mean difference (MD) or standard mean difference (SMD) was used to pool the results for continuous variables, such as duration of surgery and blood loss. The significance level for all analyses was set at *P* < 0.05. Heterogeneity was assessed using the I^2^ statistic. A fixed-effects model was utilized for each outcome where I^2^ < 50% and a random-effects model where I^2^ > 50%. Subgroup analysis was based on type of the accelerometer-based devices. Because of the small number of included studies, funnel plots were not employed to assess publication bias for all outcomes. Statistical analysis was performed using Review Manager Version 5.3.3.

## Results

### Study selection

**Fig. 1:**
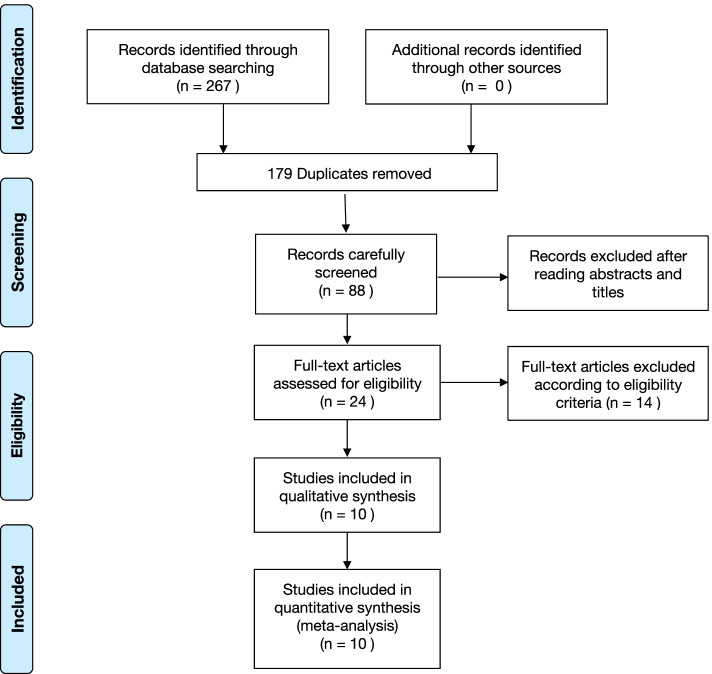
PRISMA flow chart describing the selection of articles at each stage of the process

A flow chart describes the inclusion and exclusion of studies (Fig. 1). The search yielded a total of 267 relevant articles, of which 88 remained after removal of duplicates. After screening titles and abstracts against the inclusion criteria, 24 studies were included, of which 14 were eliminated according to the exclusion criteria. Ultimately, ten studies were included in the review and meta-analysis after reading the full text of the articles.

### Patient characteristics in studies included in the review

The characteristics of patients in studies included in the review are presented in Table 1. A total of 1,125 knees treated by TKA were included in the meta-analysis, of which 573 were in the ABN group and 552 were in the CONV group. The mean age of the ABN group was 70.33 years and 70.06 years for the CONV group. Different accelerometer-based navigation systems were utilized in the different studies. Four studies used the iASSIST navigation system, two used KneeAlign, three used KneeAlign 2, while one study performed TKA with the i-Join system.

**Table 1:** Characteristic of included studies

Author	Year	Country	Implant	Device	Number		Age		BMI		Outcomes
					ABN	CONV	ABN	CONV	ABN	CONV	
Ali	2021	Australia	Legion (Smith and Nephew)	KneeAlign 2	81	78	68.1	68.2	NA	NA	KOOS WOMAC Complications
NCT03111407	2021	Spain	NA	iASSIST	44	26	67.3 ± 7.9	70.3 ± 7.4	30.4 ± 5.5	30.7 ± 4.6	MA KSS EQ-5D Complications
Kosuke	2021	Japan	Persona (Zimmer)	iASSIST	42	41	73.3 ± 9.0	76.1 ± 9.0	NA	NA	KSS OKS EQ-5D CFA CTA TS OT BL
Minoda	2020	Japan	Vanguard (Zimmer)	KneeAlign 2	45	45	76 ± 5	74 ± 7	26.5 ± 4.4	27.4 ± 4.2	MA CFA CTA OT KSS EQ-5D
Xu	2019	China	Genesis II (Smith and Nephew)	i-Join	39	40	65.3 ± 6.8	65.3 ± 7.6	NA	NA	MA CFA OT BL Complications
Kinney	2018	America	Persona (Zimmer)	iASSIST	25	25	66.4 ± 2.3	65 ± 2.0	30.4 ± 1.2	31.1 ± 1.2	MA CFA CTA OT BL Complications
Ikawa	2017	Japan	Vanguard (Zimmer)	KneeAlign 2	121	120	74 ± 6.8	74.1 ± 6.8	26.1 ± 3.7	26.8 ± 4.1	MA CFA OT BL
Gharaibeh	2017	Australia	Legion (Smith and Nephew)	KneeAlign	89	90	69.2 ± 8.7	69 ± 8.3	29.6 ± 5.4	29.2 ± 4.8	MA CFA CTA TS
Thiengwittayaporn	2016	Thailand	NexGen HiFlex (Zimmer)	iASSIST	40	40	68.0 ± 8.0	65.9 ± 6.3	26.6 ± 3.7	26.6 ± 3.7	MA CFA CTA TS OT BL
Nam	2014	America	NA	KneeAlign	47	47	67.1 ± 7.5	66.1 ± 10.1	31.1 ± 5.9	31.2 ± 5.6	MA CFA CTA TS

*KOOS* Knee Injury and Osteoarthritis Outcome Score, *WOMAC* The Western Ontario and McMaster Universities Osteoarthritis Index, *MA* Mechanical alignment, *CFA* Coronal femoral axis, *CTA* Coronal tibial axis, *TS* Tibial Slope, *EQ-5D* EuroQol five dimensions questionnaire, *OT* Operation time, *BL* Blood loss

### Risk of bias

**Fig. 2:**
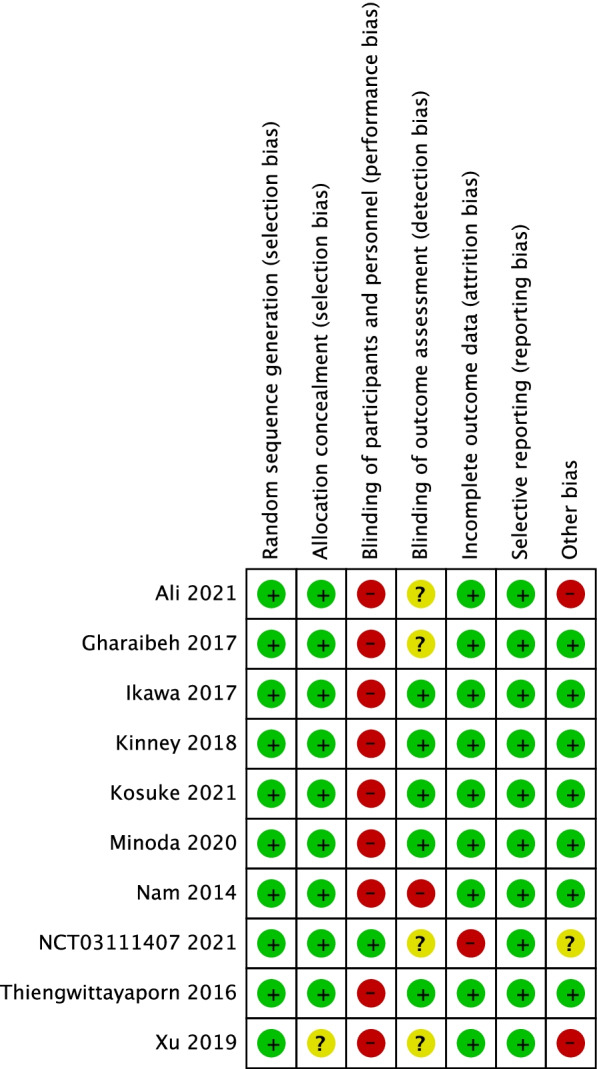
Assessment of risk of bias for each article

**Fig. 3:**
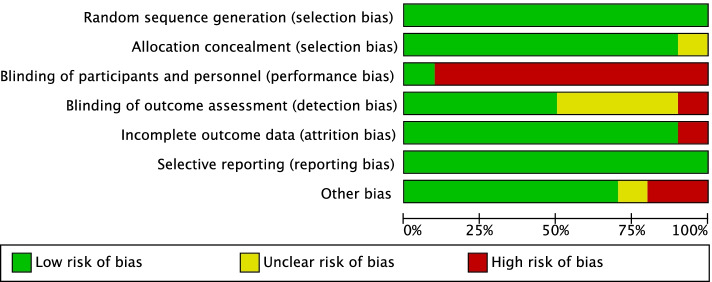
Overall quality grade of included studies

The results of the assessment are listed in Fig. 2. The type of surgery considered here prevented blinding of the surgeon to the procedure performed. All studies were considered to be at high risk for performance bias, although each was able to report the method used for random sequence generation. Therefore, we suggest that there were no incomplete outcomes or selective reporting. The overall quality of included studies is given in Fig. 3.

### Primary outcomes

**Fig. 4:**
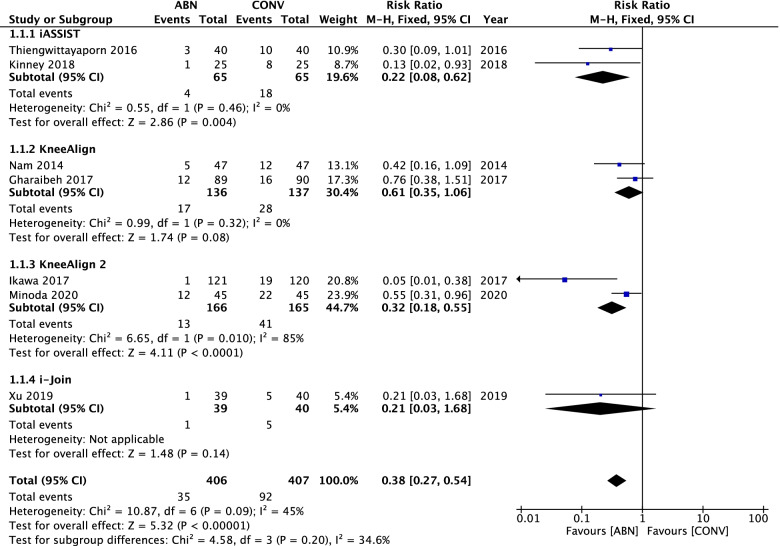
Forest plot of mechanical alignment angle (MA) outliers

**Fig. 5:**
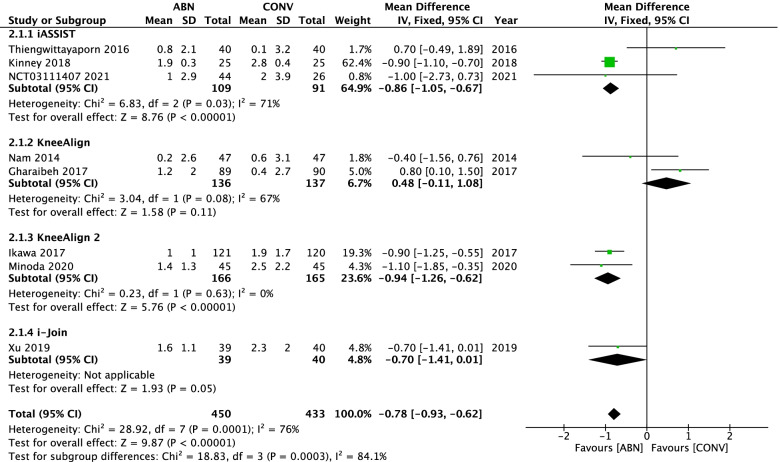
Forest plot of mechanical alignment angle (MA)

Seven studies directly compared outliers of MA for ABN and CONV. The pooled data indicated that the ABN technique significantly reduced the proportion of MA outliers compared with CONV (RR: 0.38, 95% CI: 0.27 to 0.54, *P* < 0.00001, I^2^ = 45%) (Fig. 4). Eight articles reported the mechanical alignment angle, and the results demonstrated that ABN could provide more accurate lower limb reconstruction (RR: **–**0.78, 95% CI: **–**0.93 to **–**0.62, *P* < 0.00001, I^2^ = 76%) (Fig. 5). We found that the KneeAlign group was the cause of heterogeneity, since, after exclusion of this group, I^2^ dropped from 76 to 33%.

**Fig. 6:**
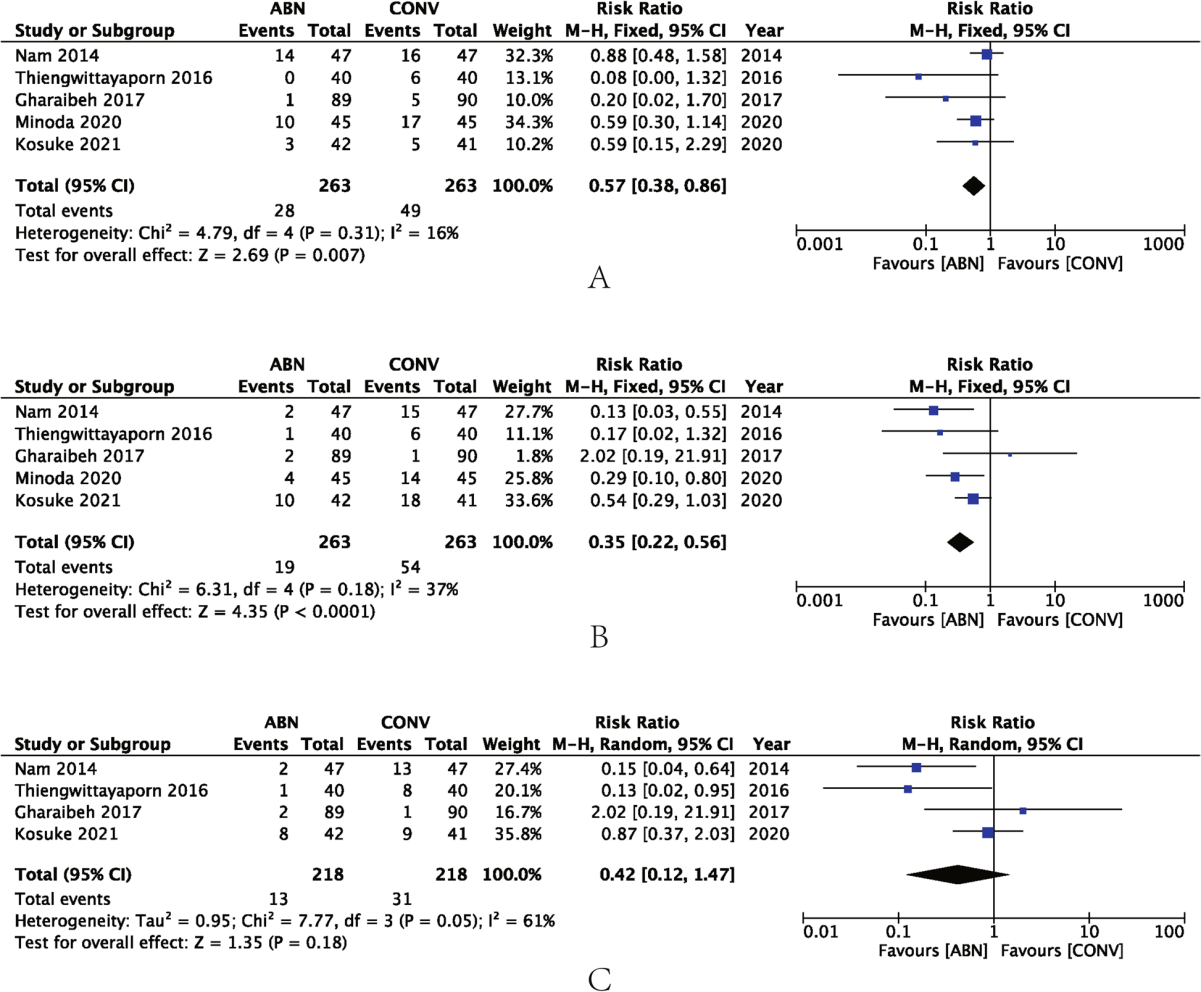
**A** Forest plot of coronal femoral angle (CFA) outliers; **B** Forest plot of coronal tibial angle (CTA) outliers; **C** Forest plot of tibial slope (TS) outliers

Five studies reported outliers for CFA, while the meta-analysis demonstrated that fewer outliers resulted in the ABN group (RR: 0.57, 95% CI: 0.38 to 0.86, *P* < 0.007, I^2^ = 16%) (Fig. 6A). The same five studies also reported outliers for CTA, the results demonstrating that CTA outliers were also decreased in the ABN group (RR: 0.35, 95% CI: 0.22 to 0.56, *P* < 0.0001, I^2^ = 37%) (Fig. 6B). Tibial slope (TS) was believed to play an important role in maintaining the sagittal axis. Four studies reported the TS and the meta-analysis showed that there was no benefit from ABN (RR: 0.42, 95% CI: 0.12 to 1.47, *P* = 0.18, I^2^ = 61%) (Fig. 6C).

### Secondary outcomes

**Fig. 7:**
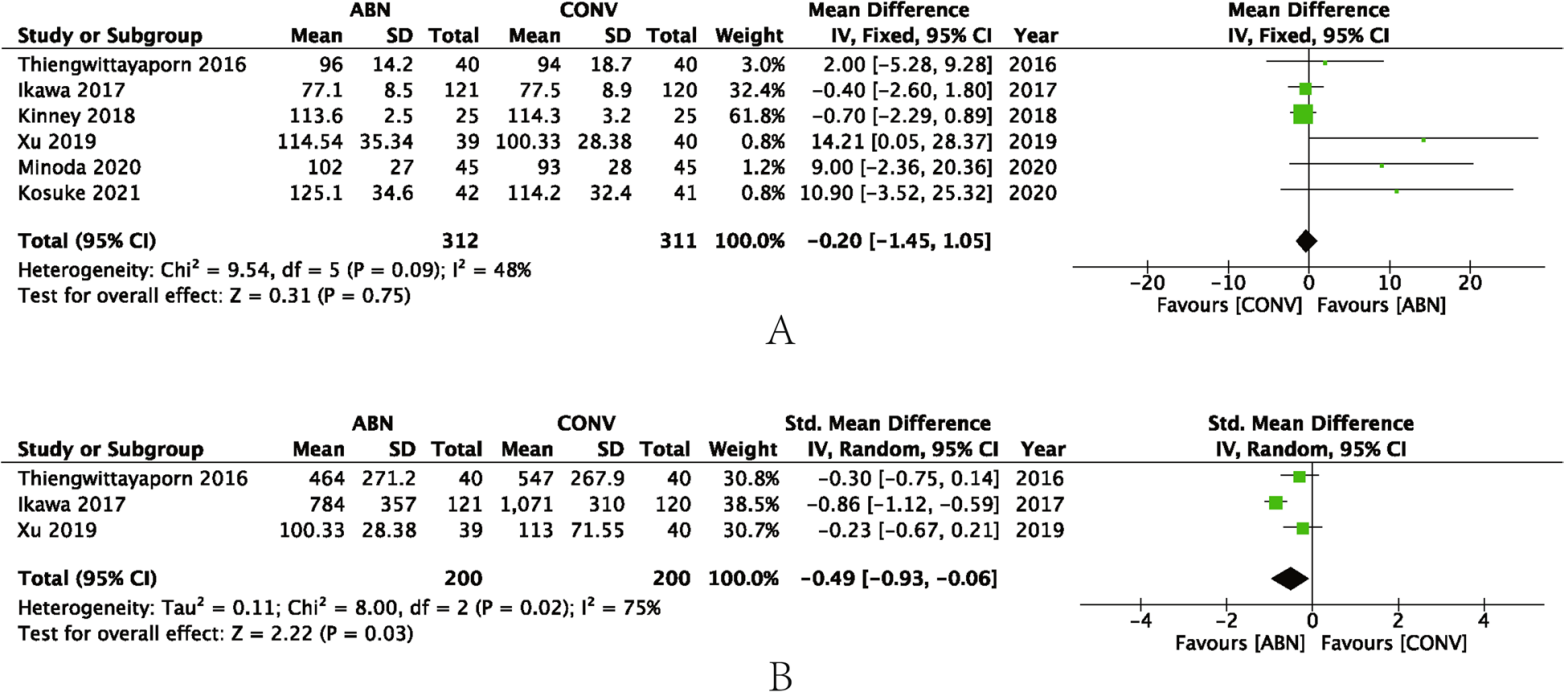
**A** Forest plot of duration of surgery; **B** Forest plot of blood loss

Six studies reported the duration of surgery as a parameter demonstrating the suitability of the ABN procedure. The pooled results revealed that there was no significant difference between the ABN and CONV groups (MD: **–**0.2, 95% CI: **–**1.45 to 1.05, *P* = 0.75, I^2^ = 48%) (Fig. 7A). Three studies reported blood loss, and, as a result of using a tourniquet or not, the volume varied greatly. SMD was used to evaluate intraoperative bleeding. After pooling the results, the data indicated the ABN group had less blood loss (SMD: **–**0.49, 95% CI: **–**0.93 to **–**0.06, *P* = 0.03, I^2^ = 75%) (Fig. 7B).

### Other outcomes

**Fig. 8:**
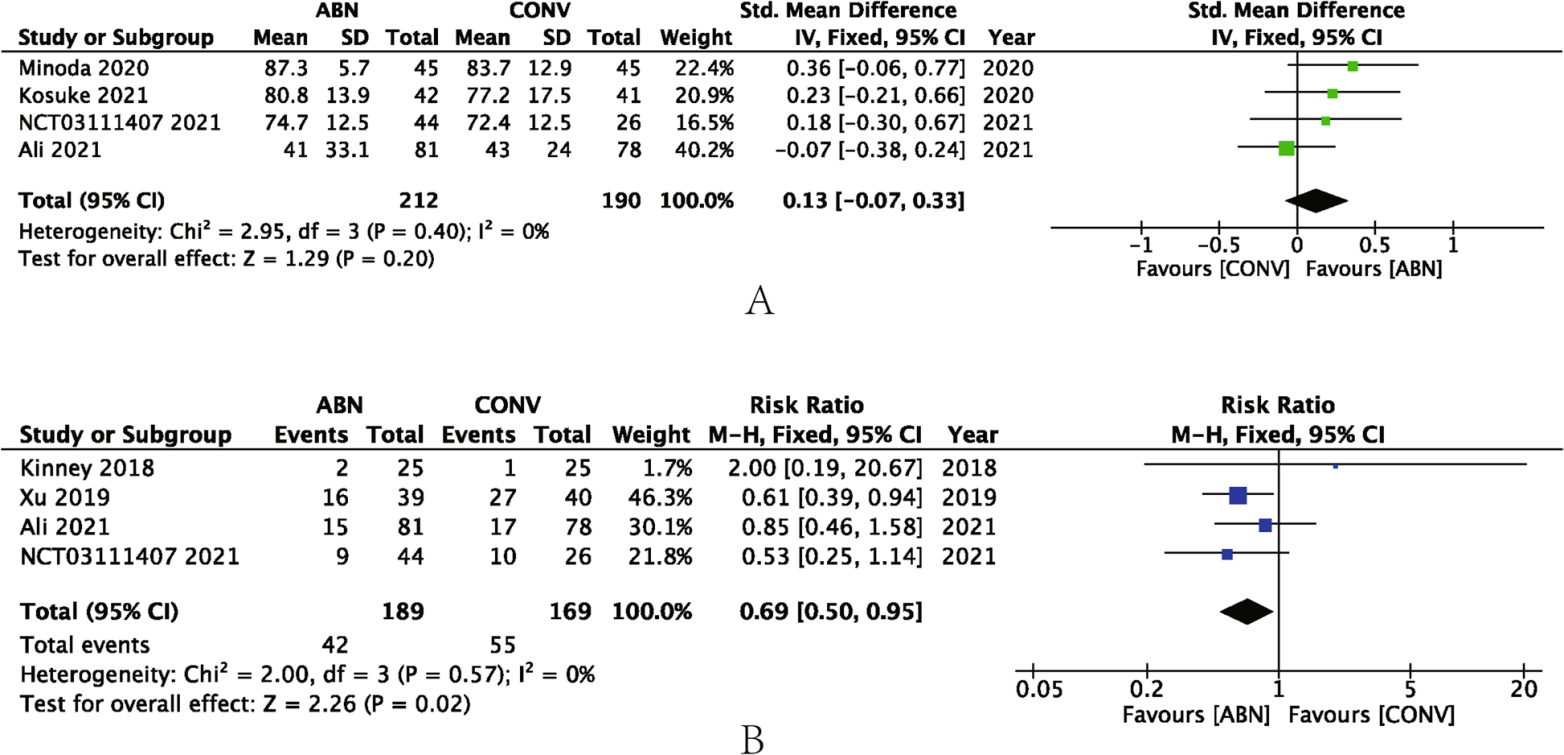
**A** Forest plot of PROMs; **B** Forest plot of overall complications

Four studies reported PROMs, for which the average follow-up time lasted for 2 years. Due to different PROMs in each study, SMD was employed in this meta-analysis, demonstrating that the ABN group did not display superior knee function compared with the CONV group (SMD: 0.13, 95% CI: **–**0.07 to 0.33, *P* = 0.20, I^2^ = 0%) (Fig. 8A). The data of overall complications were also pooled from the included studies. The results from four studies demonstrated that there was a significant difference between the ABN and CONV groups (RR: 0.69, 95% CI: 0.50 to 0.95, *P* = 0.02, I^2^ = 0%) (Fig. 8B).

## Discussion

The present meta-analysis considered 10 RCTs that assessed 1,125 knees and directly compared the clinical effectiveness of ABN with CONV for TKA. The pooled data indicated that ABN significantly reduced the proportion of outliers for MA, CFA, and CTA, and decreased blood loss during surgery. No differences were found between the two groups in terms of TS, duration of surgery, or PROMs. The use of ABN reduced the incidence of overall complications.

CONV relies on an intramedullary guiding system for cutting the bones, but many factors may influence the use of such a system, including deformity of the distal femur, obesity, and the diameter of the canal, all of which would lead to malalignment of the lower limb and an unsatisfactory outcome. In addition, intra-marrow penetration causes increased bleeding and a risk of decreased levels of hemoglobin. Computer-assisted navigation was introduced to avoid these problems. Previous studies have found that CAS decreased the risk of malalignment [29] while data from the Australian Orthopedic Association National Joint Replacement Registry has indicated that a lower revision rate was obtained by the use of CAS [30]. However, CAS has a number of shortcomings, such as complex registration, a steep learning curve, pin complications, and questionable cost-effectiveness [31, 32]. Although some CAS procedures are now pinless, the duration of surgery is significantly longer than conventional surgery [33, 34].

ABN uses a virtual framework to locate the femoral head and the center of the ankle, allowing real-time feedback to calculate the desired position [35]. Zaid Shihab *et al.* reported that ABN could attain an accuracy similar to CAS, but without requiring a skin incision or pin tracker in addition to a console outside the operative field [36]. The present study demonstrated that ABN performed well in reducing outliers on the coronal plane, with outliers for MA, CFA, and CTA being significantly decreased as compared with CONV. The proportion of outliers was similar to that reported previously [37]. Subgroup analysis of MA-related parameters suggests that, compared with conventional techniques, the effect of initial accelerometer-based navigation did not accomplish better clinical results. Conversely, iASSIST, KneeAlign 2 and i-Join navigation exhibited advantages in terms of accuracy of lower limb reconstruction. No significant difference was found in tibial slope and the test of heterogeneity demonstrated that I^2^ was 61%. Therefore, one-by-one elimination was used to assess sensitivity of the results to heterogeneity. The results indicated that there were no significant fluctuations in heterogeneity, so we believe that this result is robust.

Contrary to previous articles [36], the present review did not find any significant difference in the duration of surgery, possibly because of the simple and convenient method by which ABN is performed. As a surgeon performs more ABN procedures, she or he becomes more proficient, which flattens the learning curve. The method for locating the femoral head during ABN does not require the marrow cavity penetration, which poses risk for excessive blood loss. Different from previous meta-analyses, the present review suggests that blood loss during ABN is lower than that during CONV, which benefits postoperative recovery. We ascribed this discrepancy to the quality of the studies included in the meta-analysis. The results of the present review suggest that fewer complications occurred due to the use of ABN, and this is inconsistent with the results of previous studies. Multiple reasons might account for this result. We hypothesize that the principal reason is that the less damage was inflicted to the femoral bone marrow cavity, as compared with CONV for TKA.

Previous articles have reported that, in participants receiving TKA by CAS, only an uncertain or limited causal relationship was found for the more accurate MA and higher PROMs in 5- to 8-year follow-ups [38–40]. The present review found similar results to the studies in which CAS was used. The mean follow-up time was 2 years in the included studies, and no significant difference was found between the ABN and CONV groups. The relationship between greater accuracy and superior function remains uncertain. Sun *et al.* found that fewer outliers resulted with ABN and despite the improvement in the accuracy of component alignment, the ABN group failed to demonstrate significant superiority to the CONV group with respect to PROMs [19]. The influence of alignment is therefore debatable, and improved implant alignment due to CAS might not result in superior implant survival after 10 years [41]. The concept of kinematic alignment has become more popular in recent years, and it aims to restore native knee alignment within a pre-defined safe range. However, surgical techniques for assessing and achieving this alignment have displayed limited accuracy and reproducibility [42].

Although we conducted a comprehensive review and meta-analysis of ABN in TKA, the present review only analyzed a small number of studies. With the studies included, the sample size for a number of outcomes was relatively small, so the results may be under-powered. In addition, the follow-up period varied with the included studies, rendering reliable segmentation of results difficult, such as short-term and long-term follow-up functional results. Finally, the relationship between the accuracy of MA and implant survival remains controversial and requires a greater number of RCTs with long-term follow-up to draw any definite conclusions.

## Conclusions

The present systematic review and meta-analysis demonstrated the superiority of ABN in restoring mechanical alignment (MA) of the lower limb and improving the accuracy of the prosthetic implant. In addition, with the use of ABN, blood loss during surgery was reduced, while the duration was not prolonged. However, the present results indicate that PROMs did not improve, suggesting that superior radiographic results do not result in superior functional outcomes.

## Data Availability

The original data of this review have been uploaded to OSF (https://doi.org/10.17605/OSF.IO/AZMJE).
